# Construction of a new clinical staging system for colorectal cancer based on the lymph node ratio: A validation study

**DOI:** 10.3389/fsurg.2022.929576

**Published:** 2022-08-25

**Authors:** Yan Yang, Yawei Wang, Zhengbin Wang

**Affiliations:** ^1^ Department of General Surgery, Jiangdu People's Hospital Affiliated to Medical College of Yangzhou University, Yangzhou, China; ^2^ Department of Gastrointestinal Surgery, Clinical Medical School, Northern Jiangsu People's Hospital affiliated to Yangzhou University, Yangzhou, China; ^3^ Department of General Surgery, Jiangsu Hospital of Integrated Traditional Chinese and Western Medicine, Nanjing, China; ^4^ Department of Gastrointestinal Surgery, Affiliated Hospital of Yangzhou University, Yangzhou, China

**Keywords:** lymph node ratio, AJCC staging system, colorectal cancer, survival, CRC

## Abstract

**Aim:**

This study aims to construct a new staging system for colorectal cancer (CRC) based on the lymph node ratio (LNR) as a supplement to the American Joint Committee on Cancer (AJCC) tumor node metastasis (TNM) staging system for predicting the prognosis of CRC patients with <12 lymph nodes.

**Methods:**

The data of 26,695 CRC patients with <12 lymph nodes were extracted from the Surveillance, Epidemiology, and End Results (SEER) database as a training set. A total of 635 CRC patients were also enrolled from Northern Jiangsu People's Hospital affiliated with Yangzhou University as an independent validation set. Classification and regression tree analysis was used to obtain the LNR cutoff value. Survival curves were estimated using the Kaplan–Meier method, and the log-rank test was used for comparisons of differences among the survival curves. The monotonic decreasing trend of the overall survival curve in the staging system was expressed by the linear correlation degree R.

**Results:**

The 5-year survival rates of patients in the training set based on the AJCC staging system from stage I to stage IV were 75.6% (95%CI: 74.4–76.8), 59.8% (95%CI: 58.6–61.0), 42.1% (95%CI: 34.5–49.7), 33.2% (95%CI: 24.6–41.8), 72.0% (95%CI: 69.1–74.9), 48.8% (95%CI: 47.4–50.2), 26.5% (95%CI: 23.0–30.0), and 11.3% (95%CI: 10.3–12.3). The 5-year survival rates of patients in the training set from stage I to stage IIIC were 80.4%, 72.9%, 59.8%, 48.4%, 32.5%, and 15.0%, according to the TNM + LNR (TNRM) staging system. According to the AJCC staging system, the 5-year survival rates of patients in the validation set from stage I to stage IIIC were 91.3%, 90.8%, 72.6%, 61.3%, 72.4%, 58.1%, and 32.8%. Based on the TNRM staging system, the 5-year survival rates of patients in the validation set from stage I to stage IIIC were 99.2%, 90.5%, 81.4%, 78.6%, 60.2%, and 35.8%.

**Conclusion:**

The TNRM staging system successfully eliminated “survival paradox” in the AJCC staging system, which might be superior to the AJCC staging system.

## Introduction

Colorectal cancer (CRC) is the third most common malignancy, with the third highest mortality rate all over the world (1). The global burden of CRC has been estimated to increase by 60%, with more than 2.2 million new cases and 1.1 million cancer deaths by 2030 (2). Although the diagnosis and treatment of CRC have improved in the previous years, the 5-year survival rate for CRC was only 50% (3). Currently, the American Joint Committee on Cancer (AJCC) 8th version tumor node metastasis (TNM) staging system is the most widely used tool for predicting the survival of CRC patients (4). In general, higher-stage malignant tumors were associated with a lower survival rate (5). It is strange that a survival contradiction between stage IIIA and stage II of CRC existed in the current TNM staging system, which refers to the “survival paradox” (6, 7). Edge and Compton reported that the 5-year survival rate of CRC patients at stage IIB/C was about 46%–61%, which was lower than that of patients at stage IIIA (70%) (5). Several previous studies have shown that the prognosis of CRC patients at stage IIIA was better than patients at stages IIB and IIC (8, 9). Additionally, the existing AJCC N staging system requires at least 12 lymph nodes to be excised and examined histopathologically to ensure a reliable result (10). However, the total number of retrieved lymph nodes for CRC patients in actual clinical practice is often <12 (11). Constructing a new clinical staging system for predicting the prognosis of CRC patients with insufficient lymph nodes is of great value.

Previously, researchers have established several new staging systems combining the AJCC staging system with other biomarkers to evaluate the prognosis of CRC patients. Liu et al. combined carcinoembryonic antigen (CEA) levels with the AJCC staging system to assess the prognosis of CRC patients (12). Another study modified the pathological N stage of the AJCC, including the status of tumor deposits, to evaluate the prognosis and survival of CRC patients, especially those with positive regional lymph nodes (13). In a study by Liu et al., the prognostic score was supplemented with the AJCC TNM staging system to improve the prognostic accuracy and clinical management of colon cancer (14). These studies aimed to measure the prognosis of all CRC patients despite the number of retrieved lymph nodes. The clinical staging system for improving the prognostic accuracy of CRC patients with insufficient lymph nodes was still lacking.

Lymph node ratio (LNR), calculated as the ratio of the number of metastatic lymph nodes to the total number of lymph nodes examined, is now being proposed to address problems related to staging deviation caused by the lack of the total number of lymph nodes (15). LNR is reported to be an essential prognosis indicator of CRC patients (16). Previous studies also indicated that LNR has an advantage over the AJCC N staging system in predicting the prognosis of CRC patients with inadequate retrieved lymph nodes (17, 18). In light of these considerations, based on the data from the Surveillance, Epidemiology, and End Results (SEER) database, our study aimed to construct a new clinical staging system including LNR to supplement the existing AJCC TNM staging system (TNRM staging system) in predicting the prognosis of CRC patients with limited lymph nodes. We also used the data of CRC patients from our hospital to validate the efficiency of the TNRM staging system for predicting the prognosis of CRC patients with limited lymph nodes.

## Methods

### Study population

This study extracted the data of 135,980 CRC patients between 2004 and 2016 with <12 lymph nodes from the SEER database [SEER 18 Regs Custom Data (with additional treatment field), Nov 2018 Sub (1973–2016 varying)]. SEER is a representative program that collects demographic, clinical, and outcome information about all cancers, which covers 18 geographically diverse populations with 30% of the US population (19). The diagnosis of CRC was based on the International Classification of Diseases for Oncology, Third Edition (ICD-O-3), histology codes 8020/3, 8032/3, 8070/3, 8140/3, 8201/3, 8213/3, 8480/3, 8490/3, 8510/3, 8560/3, “Site recode ICD-O-3/WHO 2008.” After excluding patients who had nonprimary tumors and participants with unclear pathological diagnosis, invalid follow-up, no surgery, appendiceal tumor and unclear tumor location, a history of radiotherapy before and/or after operation, unclear pathological grading, unclear tumor size, unclear number of positive lymph nodes, and unclear tumor (T) and metastasis (M) stages of the AJCC 8th version, a total of 26,695 patients were eventually included in our study.

**Figure 1:**
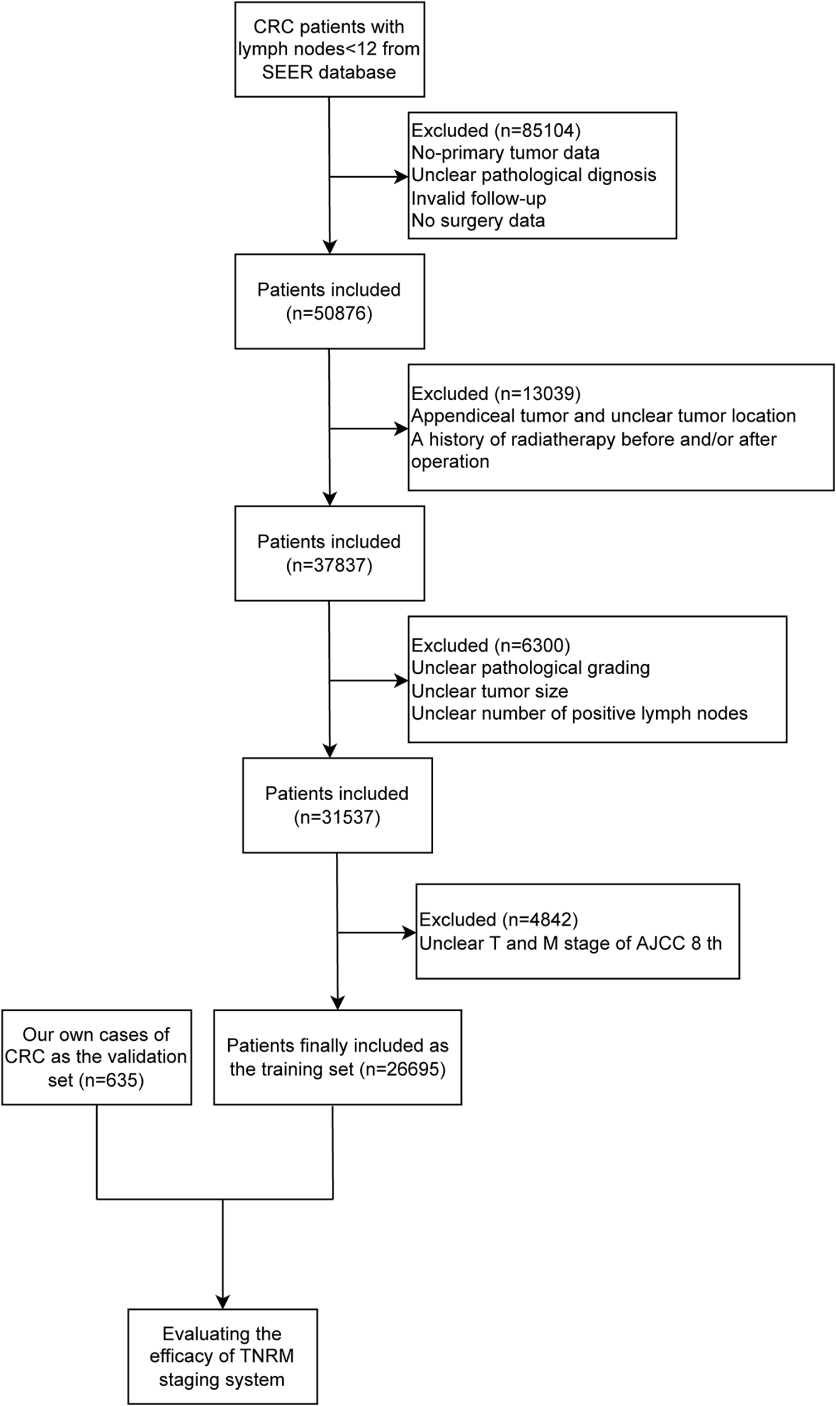
The screening process of the participants in this study.

The data of 635 CRC patients were also collected from the Gastrointestinal Center of Northern Jiangsu People's Hospital affiliated with Yangzhou University between 2012 and 2016 as an independent validation set. The study was approved by the Ethics Committee of the Northern Jiangsu People's Hospital affiliated with Yangzhou University (2019081). All patients were identified by pathological diagnosis, and patients with missing data on T, node (N), or M staging or survival were excluded. The detailed screening process of all participants from the SEER database and our own cases is shown in Figure 1.

### Data collection

The clinical data of 26,695 CRC patients from the SEER database and 635 CRC patients from Northern Jiangsu People's Hospital affiliated with Yangzhou University were collected, including the mean age at diagnosis (≥60 or <60 years), marital status (married or other), sex, race (White or not White), tumor site (colon or rectum), pathological grade (I/II or III/IV), tumor size (≤5 or >5 cm), AJCC T stage (T1, T2, T3, T4a, or T4b), AJCC N stage (N0, N1a, N1b/c, N2a, or N2b), AJCC M stage (M0 or M1), CEA (elevated or normal/unknown), perineural invasion (positive or negative/unknown), and chemotherapy (yes or no/unknown).

### Comparisons of different versions of AJCC TNM staging

In terms of the comparison of the AJCC 6th and 7th versions, the main substaging remained unchanged in the 7th version. Some N2 diseases were reclassified from stage IIIC to stage IIIA/IIIB, and stage IIB (T4N0) disease was further subdivided into stages IIB (T4aN0) and IIC (T4bN0). Subdivision of N stages was made in the 7th version of AJCC, including N1 into N1a and N1b, N2 into N2a, N2b, and a new definition of N1c for tumor deposits. M stage was changed, including M1a for single-site metastasis and M1b for multiple-site or peritoneal metastasis.

The AJCC 8th version added stage IVC, which referred to patients at a stage involving peritoneal metastasis with or without metastasis of other organs. The 8th version of AJCC continues to use vascular lymphatic vessel infiltration and tumor deposition as prognostic predictors, while microsatellite instability status and BRAF status are applied to evaluate prognostic risk, and BRAF, KRAS, and the degeneration of the NRAS were utilized as efficacy predictors.

### Formulation of LNR grouping

**Figure 2:**
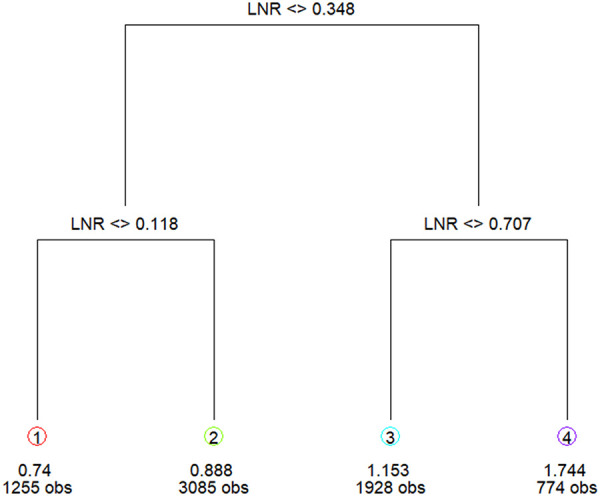
The classified regression tree analysis showed the optimal LNR cutoff value.

M1 patients with long-term metastasis (*n* = 4,694) were grouped into IV stage in accordance with the AJCC staging system as those patients could be at any N stage and thus were excluded from the LNR grouping. LNR grouping was performed based on the data of 22,001 patients with no distant metastasis. Patients with metastatic lymph nodes and no long-term metastasis (*n* = 7,404) in the training set were grouped according to the cutoff value of classification and regression tree analysis (17). Patients with metastatic lymph nodes and no long-term metastasis were included in the classified regression tree analysis to identify the optimal LNR cutoff value: LNR1: 0 < LNR ≤ 0.12; LNR2: 0.12 < LNR ≤ 0.35; LNR3: 0.35 < LNR ≤ 0.71; LNR4: 0.71 < LNR ≤ 1 (Figure 2).

Lymph node-negative patients with ≥6 lymph nodes were classified as LNR0, and the 5-year survival rate of these patients (*n* = 10,674) was significantly better than that of lymph node-negative patients with ≤5 lymph nodes (*n* = 3,747) (67.2% vs. 62.4%, *P *< 0.001). The 5-year survival rate of lymph node-negative patients with ≤5 lymph nodes was not statistically different from LNR1 patients with positive lymph nodes (62.4% vs. 59.9%, *P *= 0.203), and these two groups were combined into LNR1. The 5-year survival rates of patients at the N1c stage (*n* = 176) with tumor deposits were not statistically different with lymph node-positive LNR3 (48.0% vs. 43.7%, *P *= 0.712), and the two groups were merged into LNR3.

### Construction and validation of the TNRM staging system

The data of 135,980 CRC patients from the SEER database were regarded as the training set for constructing the TNRM staging system. LNR grouping was conducted based on the cutoff value of LNR. Univariate and multivariate Cox regression analyses were applied to identify factors associated with the prognosis of CRC. A total of 635 cases of CRC from the Gastrointestinal Center of Northern Jiangsu People's Hospital affiliated with Yangzhou University were applied for external validation of the efficacy of the TNRM staging system.

### Statistical analysis

Survival curves were estimated using the Kaplan–Meier method, and the log-rank test was used for comparisons of differences among the survival curves. Factors associated with the prognosis of CRC patients were investigated using univariate and multivariate Cox regression analyses. The monotonic decreasing trend of the overall survival (OS) curve in the staging system was expressed by the linear correlation degree R to assess and compare the prediction ability of the TNRM staging system for prognosis of CRC patients. R version 3.5.0 (R Foundation for Statistical Computing, Vienna, Austria), r-part, and map-tree software packages were applied to construct the model. SPSS version 24.0 was employed for data analyses, and the entire test was two-sided. *P *< 0.05 was considered statistically significant.

## Results

### Baseline characteristics of all participants

In total, 135,980 CRC patients with <12 lymph nodes from the SEER database were included in the current study. Among them, patients who had nonprimary tumor as well as those with unclear pathological diagnoses, invalid follow-up, and no surgery were excluded (*n* = 85,104). Patients with appendiceal tumor and unclear tumor location and history of radiotherapy before and/or after operation were also excluded (*n* = 13,039). After excluding patients with unclear pathological grading, unclear tumor size, and unclear number of positive lymph nodes (*n* = 6,300) and patients with unclear T and M stages (*n* = 4,842), 26,695 patients were finally included in our study as the training set. A total of 635 CRC patients were recruited from Northern Jiangsu People's Hospital affiliated with Yangzhou University between 2012 and 2016 and included as the validation set.

In the cohort of patients with <12 lymph nodes from the SEER database, 20,350 (76.2%) patients were ≥60 years. A total of 22,355 (83.7%) people with primary tumors in the colon, and 4,340 (16.3%) patients had tumors located in the rectum. A total of 20,520 (76.9%) patients were associated with tumors ≤5 cm. A total of 21,907 (82.1%) patients were at pathological grade I/II. A total of 17,313 (64.9%) patients were classified as postoperative stage T3, and 7,744 (29.0%) patients received chemotherapy. As for patients from Northern Jiangsu People's Hospital affiliated with Yangzhou University, 461 (72.6%) were ≥60 years. A total of 390 (61.4%) patients were male. There were 246 (38.7%) patients with the tumor site in the colon, and 389 (61.3%) patients had tumors located in the rectum. A total of 382 (60.2%) patients were at pathological grade I/II. There were 479 (75.4%) patients with tumor size >5 cm. A total of 311 (49.0%) patients received chemotherapy (Table 1).

**Table 1:** The demographic and clinical data of all patients.

Characteristic	Training set (*n* = 26,695)	Validation set (*n* = 635)
Age at diagnosis, mean (years)		
≥60	20,350 (76.2)	461 (72.6)
<60	6,345 (23.8)	174 (27.4)
Marital status		
Married	14,002 (52.5)	522 (82.2)
Other	12,693 (47.5)	113 (17.8)
Sex		
Male	13,338 (50.0)	390 (61.4)
Female	13,357 (50.0)	245 (38.6)
Race		
White	20,844 (78.1)	0 (0)
Not White	5,851 (21.9)	637 (100)
Tumor site		
Colon	22,355 (83.7)	246 (38.7)
Rectum	4,340 (16.3)	389 (61.3)
Pathological grade		
I/II	21,907 (82.1)	382 (60.2)
III/IV	4,788 (17.9)	253 (39.8)
Size, cm		
≤5	20,520 (76.9)	156 (24.6)
>5	6,175 (23.1)	479 (75.4)
AJCC T stage		
T1	2,306 (8.6)	28 (4.4)
T2	5,152 (19.3)	94 (14.8)
T3	17,313 (64.9)	66 (10.4)
T4a	1,245 (4.7)	335 (52.8)
T4b	679 (2.5)	112 (17.6)
AJCC N stage		
N0	15,399 (57.1)	384 (60.5)
N1a	3,585 (13.4)	88 (13.9)
N1b/c	4,125 (15.5)	113 (17.8)
N2a	2,500 (9.4)	37 (5.8)
N2b	1,086 (4.1)	13 (2.0)
AJCC M stage		
M0	22,001 (82.4)	630 (99.2)
M1	4,694 (17.6)	5 (0.8)
CEA		
Elevated	6,736 (25.2)	267 (42.0)
Normal/unknown	19,959 (74.8)	368 (58.0)
Perineural invasion		
Positive	1,095 (4.1)	58 (9.1)
Negative/unknown	25,600 (95.9)	577 (90.9)
Chemotherapy		
Yes	7,744 (29.0)	311 (49.0)
No/unknown	18,951 (71.0)	324 (51.0)

CEA, carcinoembryonic antigen; AJCC, The American Joint Committee on Cancer; TNM, tumor node metastasis.

### Five-year survival rate of CRC patients in the training set from the SEER database based on the AJCC TNM staging system

**Figure 3:**
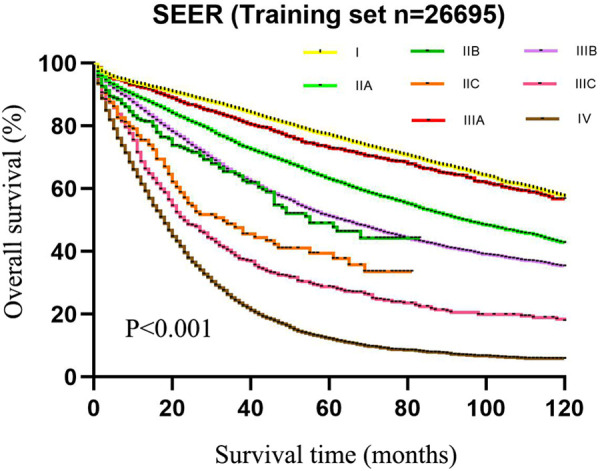
The 5-year survival rate of patients in the training set using CRC patients from the SEER database based on the AJCC TNM staging system.

As exhibited in Table 2, the 5-year survival rates in patients were 75.6% (95%CI: 74.4–76.8) at stage I, 59.8% (95%CI: 58.6–61.0) at stage IIA, 42.1% (95%CI: 34.5–49.7) at stage IIB, 33.2% (95%CI: 24.6–41.8) at stage IIC, 72.0% (95%CI: 69.1–74.9) at stage IIIA, 48.8% (95%CI: 47.4–50.2) at stage IIIB, 26.5% (95%CI: 23.0–30.0) at stage IIIC, and 11.3% (95%CI: 10.3–12.3) at stage IV. As shown in the Kaplan–Meier survival curve, the 5-year survival rate of stage IIIA was significantly higher than that of stages IIB (72.0% vs. 42.1%) and IIC (72.0% vs. 33.2%) and even better than that of stage IIA (72.0% vs. 59.8%) (Figure 3). The results illustrated that CRC patients with insufficient lymphoid examination were associated with a “survival paradox” based on AJCC staging.

**Table 2:** The 5-year survival rate of CRC patients in the training set from the SEER database based on AJCC staging.

AJCC	*n*	5-year survival rate	95%CI	*P* value
I	6,105	75.6	74.4–76.8	0.006
IIA	7,900	59.8	58.6–61.0	0.006
IIB	249	42.1	34.5–49.7	0.039
IIC	167	33.2	24.6–41.8	0.044
IIIA	1,026	72	69.1–74.9	0.015
IIIB	5,876	48.8	47.4–50.2	0.007
IIIC	678	26.5	23.0–30.0	0.018
IV	4,694	11.3	10.3–12.3	0.005

CI, Confidence interval; AJCC, The American Joint Committee on Cancer; CRC, colorectal cancer; SEER, Surveillance, Epidemiology, and End Results.

### AJCC N staging and LNR grouping for predicting the 5-year survival rate of CRC patients in the training set from the SEER database and factors associated with the survival of CRC patients

Based on the LNR grouping, the 5-year survival rates of the five groups from LNR0 to LNR4 were 67.2% (*n* = 10,674), 61.8% (*n* = 5,044), 55.2% (*n* = 3,233), 43.9% (*n* = 2,215), and 30.6% (*n* = 835), respectively. Statistical differences were obtained among the groups (*χ*^2 ^= 834.572, *P *< 0.001) (Table 3).

**Table 3:** Univariate and multivariate analyses identifying factors associated with the prognosis of patients with CRC.

Characteristics	*n*	5-year OS (%)	Univariate analysis		Multivariate analysis	
			Standard error	*P* value	HR (95%CI)	*P* value
Marital status				<0.001		<0.001
Married	11,513	67.7	0.005		1	
Other	10,488	52.6	0.005		1.393–1.503	
Age at diagnosis, mean (years)	** **			<0.001		<0.001
≥60	17,188	55.3	0.004		1	
<60	4,813	79.5	0.006		0.365–0.413	
Race				<0.001		<0.001
White	17,336	59.9	0.004		1	
Not white	4,665	62.8	0.008		0.829–0.912	
Sex				>0.05		
Male	10,882	60.3	0.005			
Female	11,119	60.7	0.005			
Tumor site				<0.001		0.891
Colon	18,312	59.9	0.004		1	
Rectum	3,689	63.6	0.008		0.953–1.057	
Pathological grade				<0.001		<0.001
I/II	18,538	62.8	0.004		1	
III/IV	3,463	48.2	0.009		1.124–1.239	
Size, cm				<0.001		<0.001
≤5	17,543	63.6	0.004		1	
>5	4,458	48.1	0.008		1.208–1.321	
Chemotherapy				<0.001		<0.001
Yes	5,061	68.2	0.007		1	
No/unknown	16,940	58.2	0.004		1.905–2.119	
CEA				<0.001		<0.001
Elevated	4,199	49	0.008		1	
Normal/unknown	17,802	63	0.004		0.772–0.846	
Perineural invasion						<0.001
Positive	548	37	0.026	<0.001	1	
Negative/unknown	21,453	61	0.003		0.650–0.827	
AJCC T stage				<0.001		<0.001
T1	2,271	80.1	0.009		1	
T2	4,993	72.3	0.007			
T3	13,730	54.9	0.004			
T4a	667	34.2	0.023			
T4b	340	24	0.028			
N stage				<0.001		0.119
N0	14,421	66	0.004		1	
N1a	2,811	56.4	0.01			
N1b/c	2,885	51.6	0.01			
N2a	1,420	41	0.014			
N2b	464	28.5	0.022			
LNR grouping				<0.001		<0.001
LNR0	10,674	67.2	0.005		1	
LNR1	5,044	61.8	0.007			
LNR2	3,233	55.2	0.009			
LNR3	2,215	43.9	0.011			
LNR4	835	30.6	0.017			

HR, hazard ratio; CEA, carcinoembryonic antigen; AJCC, The American Joint Committee on Cancer; TNM, tumor node metastasis; CRC, colorectal cancer; OS, overall survival; LNR, lymph node ratio.

The 5-year survival rates of patients based on the AJCC N staging system were 66.0% (*n* = 14,421) at N0, 56.4% (*n* = 2811) at N1a, 51.9% (*n* = 2709) at N1b, 48.0% (*n* = 176) at N1c, 41.0% (*n* = 1420) at N2a, and 28.5% (*n* = 464) at N2b (*χ*^2 ^= 698.650, *P *< 0.001). The survival rates between N1b and N1c were not statistically different (*χ*^2 ^= 2.586, *P *= 0.108), and patients with N1c and N1b were combined into one group. The 5-year overall survival rates of patients were 66.0% (*n* = 14,421) at N0, 56.4% (*n* = 2811) at N1a, 51.6% (*n* = 2885) at N1b/c, 41.0% (*n* = 1420) at N2a, and 28.5% (*n* = 464) at N2b. The difference in the survival rates among the five groups was statistically different (*χ*^2 ^= 694.258, *P *< 0.001) (Table 3).

The results of the univariate analysis revealed that AJCC N stage (*P *< 0.001) and LNR grouping (*P *< 0.001), age (*P *< 0.001), tumor location (*P *< 0.001), tumor size (*P *< 0.001), tumor pathological grade (*P *< 0.001), tumor T stage (*P *< 0.001), and chemotherapy (*P *< 0.001) were associated with the prognosis of patients. Factors with a statistical difference were included in the multivariate analysis, which showed that the AJCC N stage (*P *> 0.05) was not statistically associated with the prognosis of patients, while the LNR grouping (*P *< 0.001) remained as an independent factor associated with the survival rate of patients (Table 3).

### Construction of a new staging system based on the AJCC T stage and LNR grouping

The new staging system was established based on the combination of the AJCC T stage and LNR grouping. The training set was divided into a total of 25 different groups obtained according to five T stages and five LNR groupings (Table 4). The 5-year survival rate of each stage was calculated, and the survival curves were compared pairwise. The new groups were sorted according to the 5-year survival rate. Neighborhood survival curves with no statistical difference were combined, and three new stage groups were formed:

1. good prognosis stage with a 5-year survival rate ≥75% was classified as stage I;
2. moderate prognosis stage with a 5-year survival rate of 55%–75% was classified as stage II; and
3. poor prognosis stage with a 5-year survival rate <55% was classified as stage III.

**Table 4:** The new staging system based on the AJCC T stage and LNR grouping.

New stage	Combination serial number	Phased combination	*n*	5-year OS (95CI) (%)
I	1	T1LNR0	1359	82.6 (80.4–84.8)
	6	T1LNR1	731	76.0 (72.9–79.1)
	11	T1LNR2	125	76.0 (68.2–83.4)
IIA	2	T1LNR3	2999	74.3 (72.7–75.9)
	12	T2LNR2	454	72.5 (68.2–76.8)
	16	T1LNR3	47	71.0 (57.3–84.7)
	7	T2LNR1	1277	70.0 (67.5–72.5)
IIB	17	T2LNR3	209	62.0 (54.9–69.1)
	3	T3LNR0	6022	61.4 (60.0–62.7)
	8	T3LNR1	2827	56.2 (54.2–58.2)
IIIA	13	T3LNR2	2455	52.5 (50.5–54.5)
	21	T1LNR4	9	47.6 (10.9–84.3)
	22	T2LNR4	54	46.5 (32.8–60.2)
	4	T4aLNR0	175	46.0 (37.2–54.8)
	18	T3LNR3	1757	42.6 (40.2–45.0)
IIIB	14	T4aLNR0	155	37.0 (27.6–46.4)
	5	T4bLNR0	119	35.5 (25.5–45.5)
	19	T4aLNR3	132	34.5 (24.7–44.3)
	23	T3LNR4	669	32.0 (28.3–35.7)
	9	T4aLNR1	136	25.5 (15.3–35.7)
IIIC	10	T4bLNR1	73	23.0 (11.0–35.0)
	15	T4bLNR2	44	19.7 (6.0–33.4)
	24	T4aLNR4	69	18.0 (8.2–27.8)
	20	T4bLNR3	70	13.5 (0.17–26.8))
	25	T4bLNR4	34	3.5 (0–10.2))

LNR, lymph node ratio; CI, confidence interval; OS, overall survival; AJCC, The American Joint Committee on Cancer.

Stage III was further classified into IIIA with a 5-year survival rate of 40%–55%, IIIB with a 5-year survival rate of 25%–40%, and IIIC with a 5-year survival rate <25% (Table 4). Finally, six new TNRM staging were obtained (Table 5).

**Table 5:** TNRM staging system (suitable for LNs < 12).

TNRM	T1	T2	T3	T4a	T4b
LNR0	I	IIA	IIB	IIIA	IIIB
LNR1	I	IIA	IIB	IIIB	IIIC
LNR2	I	IIA	IIIA	IIIB	IIIC
LNR3	IIA	IIB	IIIA	IIIB	IIIC
LNR4	IIIA	IIIA	IIIB	IIIC	IIIC

LNR, lymph node ratio; TNRM, TNM + LNR; LN, lymph node.

### Efficiency of the TNRM staging system for predicting the 5-year survival rate of CRC patients in the training set from the SEER database

**Figure 4:**
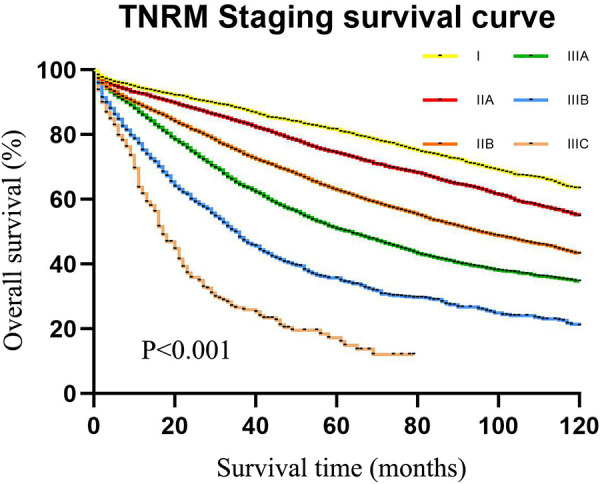
The 5-year survival rate of CRC patients in the training set using CRC patients from the SEER database according to TNRM staging.

As displayed in Figure 4, the 5-year survival rates of patients from stage I to IIIC were 80.4%, 72.9%, 59.8%, 48.4%, 32.5%, and 15.0%, according to the TNRM staging system (*χ*^2 ^= 1765.947, *P *< 0.001).

The results of the chi-square test displayed that the TNRM staging system monotonously decreased in the whole interval with an R value of 1215.621, while the R value was 653.858 for the AJCC staging system, which revealed that the new TNRM staging system was also superior to the AJCC system in the monotonic decreasing gradient of each substage.

The AJCC TNM stages were restratified by TNRM stages to compare the homogeneity of the TNRM and AJCC TNM staging systems. As shown in Table 6, the AJCC IIIC stage was found to be the most heterogeneous in respect of survival; it contained three new TNRM stages as follows: IIIA (*n* = 85), IIIB (*n* = 363), and IIIC (*n* = 230) with the 5-year OS ranging from 10.3% to 45.0% (range = 34.7%). The survival span of the AJCC IIIB stage (*n* = 5836) containing three TNRM subgroups also showed poor homogeneity with a 5-year OS of 18.0%–58.9% (range = 30.9%). When TNRM stages (stages I–IIIC) were restratified according to AJCC stages, the most heterogeneity was found in the TNRM IIIC stage with a survival span of 10.3%–27.6% (range = 17.3%), and the sample size in this stage was very small (*n* = 290).

**Table 6:** Five-year overall survival by AJCC staging and TNRM staging for predicting the survival of CRC patients in the training set from SEER.

AJCC stage (*n*)	TNRM stage					
	I, 80.4% (2215)	IIA, 72.9% (4777)	IIB, 59.8% (9058)	IIIA, 48.4% (4450)	IIIB, 32.5% (1211)	IIIC,14.8% (290)
I, 75.6% (6105)	80.5% (2036)	73.1% (4069)				
IIA, 59.8% (7900)			59.8% (7900)			
IIB, 42.3% (249)				46.0% (175)	33.5% (74)	
IIC, 33.5% (167)					35.5% (119)	27.6% (48)
IIIA, 72.0% (1026)	78.0% (179)	71.8% (708)	68.0% (104)	52.5% (35)		
IIIB, 48.8% (5836)			58.9% (1054)	48.4% (4115)	33.6% (655)	18.0% (12)
IIIC, 26.4% (678)				45.0% (85)	30.0% (363)	10.3% (230)

AJCC, The American Joint Committee on Cancer; TNRM, TNM + LNR; CRC, colorectal cancer; SEER, Surveillance, Epidemiology, and End Results.

### External validation of the TNRM staging system of the 5-year survival rate of our CRC cases

**Figure 5:**
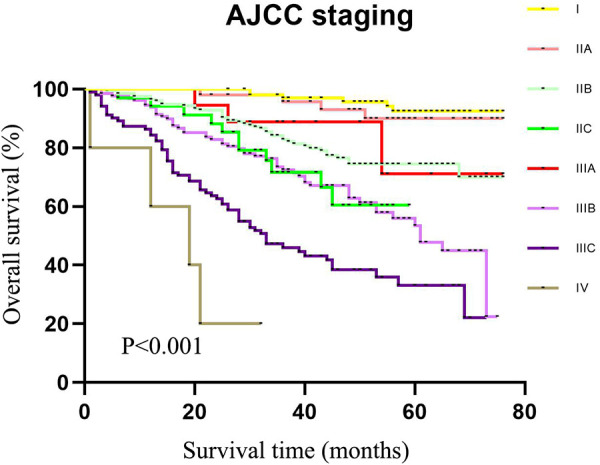
The 5-year survival rate of patients in the validation set using our cases of CRC based on the AJCC TNM staging system.

**Figure 6:**
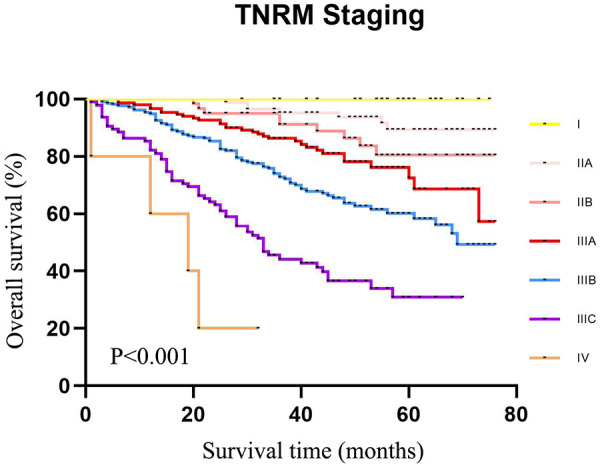
The 5-year survival rate of CRC patients in the validation set using our cases of CRC according to TNRM staging.

The data of our CRC cases from Northern Jiangsu Hospital affiliated with Yangzhou University were applied for the external validation of the performance of the TNRM staging system for predicting the 5-year survival rate of CRC patients. The survival curves of patients based on the AJCC staging system (*χ*^2 ^= 156.223, *P *< 0.001) and the TNRM staging system (*χ*^2^ = 150.648, *P *< 0.001) are shown in Figures 5 and 6, respectively.

According to the AJCC staging system, the 5-year survival rates of patients from stage I to stage IIIC were 91.3%, 90.8%, 72.6%, 61.3%, 72.4%, 58.1%, and 32.8%,. The survival rate of patients at stage IIIA was not significantly different between IIB and IIIA (72.6% vs. 72.4%, *χ*^2 ^= 0.135, *P *= 0.713). No significant difference was observed between IIC and IIIBC (61.3% vs. 58.1%, *χ*^2 ^= 0.010, *P *= 0.920). Although the sample size of AJCC stage IIIA was small, an obvious “survival paradox” was observed from the survival curves (Figure 5). Based on the TNRM staging system, the 5-year survival rates of patients from stage I to stage IIIC were 99.2%, 90.5%, 81.4%, 78.6%, 60.2%, and 35.8%. Although there was no significant difference between the survival rates at TNRM stage IIB and stage IIIA (81.4% vs. 78.6%, *χ*^2 ^= 2.026, *P *= 0.155), there was a significant difference between the survival rates at stage IIIA and other staging curves (*P *< 0.05) (Figure 6). In particular, the staging curves of the high-risk stages IIIB and IIIC were both clearly differentiated from all other stages (*P *< 0.01). In addition, the monotonic decreasing linear correlation in the TNRM staging system (*R* = 107.083) was better than that in the AJCC staging system (R = 99.188) even with one fewer staging curve (7 vs. 8).

## Discussion

The present study collected the data of 26,695 CRC patients to construct a novel clinical staging system for predicting the survival rates of CRC patients with insufficient lymph nodes *via* combining LNR ratio and AJCC T stages and validated the results in 635 of our cases of CRC from Northern Jiangsu People's Hospital affiliated with Yangzhou University. The results depicted that the 5-year survival rates of patients from stage I to stage IIIC were 80.4%, 72.9%, 59.8%, 48.4%, 32.5%, and 15.0%in the training set and 99.2%, 90.5%, 81.4%, 78.6%, 60.2%, and 35.8% in the validation set according to the TNRM staging system, which eliminated the “survival paradox” in survival rates of CRC patients estimated by the AJCC staging system. The findings of our study might provide a new tool for predicting the survival of CRC patients with <12 lymph nodes.

Previously, a study identified that the overall survival rate of CRC patients at the IIB/C stage was lower than that at the IIIA stage based on the AJCC staging system (2). In our study, we also found that for CRC patients with <12 lymph nodes examined, the overall prognosis of stage IIIA patients was better than that of stage II patients based on the AJCC staging system. Even the 5-year survival rate of CRC patients at stage IIIB was better than those at stage IIC. The results of our study indicated that the “survival paradox” of CRC patients existed in both patients with ≥12 and <12 lymph nodes examined. Some studies demonstrated that the “survival paradox” of CRC patients based on the AJCC staging system might result from stage migration or lack of systematic treatment (9). Other studies also revealed that advances in adjuvant chemotherapy and radiotherapy have improved the survival rate of CRC patients at stage III, and the benefits of adjuvant therapy may narrow the survival gap between patients at stage II and stage III (20, 21). However, for rectal cancer patients at stage II or stage III with the same adjuvant chemotherapy and radiotherapy, the prognosis of patients at stage IIIA was still better than that of patients at stage II (8). These results suggested that the “survival paradox” of CRC was not caused by adjuvant chemotherapy (22).

Li et al. suggested that the possible reason for the “survival paradox” of CRC was due to the excessive predictive weight of the N stage, and they proposed a new system by strengthening the weight of the T stage and modifying the corresponding relationship between the T stage and TN score (the T and N stage relative weights) (23). They reclassified T4bN0 into colon cancer stage IIIA and rectal cancer stage IIIB, while the AJCC stage IIIA (T1-2N1 and T1N2a) was reclassified into stage I or stage II. However, this classification also had limitations. Increasing T stages’ weight had a direct impact on the TN score. As a result, T2N2a patients with higher TN scores were classified into stage IIIA, but the 5-year overall survival rate was 66.6%, which was significantly higher than the rest of the patients in the same IIIA group. On the other hand, T1N2a patients with lower TN scores were classified into stage II, and their 5-year overall survival rate (57.4%) was significantly lower than that of the rest of stage II patients. The author explained that the inconsistency might be due to the application of linear regression in this study, but the impact of T stage and N stage combination on survival may be nonlinear. Additionally, whether the accuracy of N stage weight was affected by the number of lymph nodes examined was not investigated in the study.

In the past three decades, the AJCC TNM staging system has updated many versions. The AJCC 7th edition improved the N staging *via* dividing N1 into N1a (1 lymph node metastasis), N1b (2–3 lymph nodes metastasis), and N1c (0 lymph node metastasis) and N2 into N2a (4–6 lymph nodes metastasis) and N2b (>7 lymph nodes metastasis), and these were included in the AJCC 8th edition (24). The AJCC N stage recommends a minimum of 12 lymph nodes dissection, but the extent of lymph node dissection and the number of lymph nodes pathologically examined vary in different institutions and regions (25). At present, there are still a large number of CRC patients with <12 lymph nodes dissection (26). In our study, the median number of lymph nodes dissection was eight in patients from the training set and seven in patients from the validation set. In previous studies, the staging bias caused by insufficient lymph node dissection has been largely reported (27, 28). LNR was reported as an important prognostic predictor of CRC patients, which was not affected by the total number of lymph nodes examined (29). Currently, the stratification method and the cutoff value of LNR are still not unified, and some studies stratified LNR *via* average, quartile, receiver operator characteristic curve method, or arbitrary classification (30, 31). Different stratification methods may result in different results of the predictive value of LNR in the prognosis of CRC patients (32). Therefore, the optimal grouping standard or the cutoff value of LNR may need to be adjusted accordingly. In the present study, the LNR was grouped using classification and regression tree analysis based on Rosenberg et al. (33), who divided 3,026 patients into five groups with LNR cutoff values of 0.17, 0.41, and 0.69. They demonstrated that the cutoff values had the highest survival impact and LNR could be used as an independent prognostic factor in predicting the survival rate of CRC patients. In this study, the cutoff values of LNR were 0.12, 0.35, and 0.71 in the training set, which were close to the results from Rosenberg et al. Additionally, the lymph node-negative group was further refined. Significant differences were found in the 5-year survival rate between lymph node-negative patients with ≥6 and <6 lymph nodes. Lymph node-negative patients with ≥6 lymph nodes were included in LNR0 to optimize intrastage homogeneity (34).

Accurate cancer staging is essential for clinicians to predict the prognosis of patients, provide appropriate interventions for those patients, and choose the most effective treatment (35). Patients at stage I or stage II with a low risk of recurrence do not require adjuvant chemotherapy, while stage III patients are strongly recommended to receive adjuvant chemotherapy after surgery (36). The “survival paradox” may result in an overestimation of the prognostic risk of stage IIIA and an underestimation of the risk of stage II, which may lead to inappropriate treatments for those patients. In the current AJCC staging system, the relatively high weight of the N stage over the T stage may lead to poor monotonicity of the gradient from early stage tumor to advanced stage tumor (37). This supported the findings of our study, which revealed that the 5-year survival rates from stage I to stage IIIC in the training set were 75.6%, 59.8%, 42.1%, 33.2%, 72.0%, 48.8%, and 26.5% and in the testing set were 91.3%, 90.8%, 72.6%, 61.3%, 72.4%, 58.1%, and 32.8%. The results indicated that the “survival paradox” also existed in our study. Based on TNRM staging system, the 5-year survival rates of the training set from stage I to stage IIIC were 80.4%, 72.9%, 59.8%, 48.4%, 32.5%, and 15.0% and in the testing set were 99.2%, 90.5%, 81.4%, 78.6%, 60.2%, and 35.8%. The results demonstrated that the “survival paradox” was successfully avoided.

At present, several new staging systems combining the AJCC with other biomarkers including carcinoembryonic antigen levels, pathological N stage, and the prognostic score for evaluating the prognosis of CRC patients were established (12–14). These staging systems were applied for all CRC patients rather than CRC patients with <12 lymph nodes. No special attention was paid to those with <12 lymph nodes. The combined biomarkers such as the prognostic score were not comprehensive, and some important prognostic factors were not included (12). In addition, these studies were conducted based on the data from the SEER database with a large sample size, but no external data were applied to verify their results. This study initially constructed a TNRM staging system by combining LNR with the AJCC T stage for predicting the prognosis of CRC patients with <12 lymph nodes. The system successfully eliminated the “survival paradox” of the AJCC staging system. External validation in the validation set using our own cases of CRC also showed a better monotonic decreasing trend between each stage under the TNRM staging system. The finding of our study might help improve the prediction accuracy of the survival rate in CRC patients with <12 lymph nodes. Our study had several limitations. First, the details of surgical resection and adjuvant chemotherapy in patients could not be obtained from the SEER database. Second, the sample size in the validation set was small and from a single center, which might decrease the statistical power of our analysis. To enable a broader application of the TNRM staging system, optimization is required in the future. First, the TNRM staging system can be improved by better T stage, LNR, or M stage definitions or by adding other factors, such as tumor location. Second, whether the TNRM staging system was suitable for predicting CRC patients ≥12 lymph node dissection still requires validation in more studies as broader lymph node dissection and more lymph nodes analysis always lead to a higher staging accuracy (38). In the future, we will focus on exploring the prediction value of the TNRM system on the survival of CRC patients with ≥12 lymph nodes. More data will be collected from multiple centers to verify the findings of the current study.

## Conclusions

In the current study, the data of 26,695 CRC patients with <12 lymph nodes were collected to construct a new staging system for CRC patients with <12 lymph nodes and the data of 635 of our cases were applied for the validation of the new staging system. The results depicted that the TNRM staging system successfully eliminated “survival paradox” of the AJCC staging system, which might be superior to the AJCC staging system. The results of this study might highlight a new staging system for CRC patients with <12 lymph nodes, which might help improve the accuracy of the staging of CRC patients with <12 lymph nodes and give a reference for clinicians to provide appropriate treatment for those patients.

## Data Availability

Publicly available datasets were analyzed in this study. These data can be found here: https://seer.cancer.gov/.
